# A randomized controlled trial of exercise versus wait-list in chronic tennis elbow (lateral epicondylosis)

**DOI:** 10.3109/03009734.2011.600476

**Published:** 2011-10-29

**Authors:** Magnus Peterson, Stephen Butler, Margaretha Eriksson, Kurt Svärdsudd

**Affiliations:** ^1^Department of Public Health and Caring Sciences, Family Medicine and Clinical Epidemiology Section, Uppsala University, Uppsala, Sweden; ^2^Department of Surgical Sciences/Anesthesiology and Intensive Care Medicine, Uppsala University Hospital, Uppsala, Sweden

**Keywords:** Exercise, pain, RCT, tendinitis, tendinosis, tennis elbow

## Abstract

**Background:**

Chronic tennis elbow (lateral epicondylosis) is a common disorder. Like other chronic soft-tissue pain conditions it is often difficult to treat successfully. The effects of exercise have been discussed, but no convincing evidence has been put forward so far, and a simple protocol for exercise is lacking.

**Aims of the study:**

This study is a randomized, controlled, clinical trial of the effect of exercise versus expectation (wait-list) on pain, muscle strength, function, and quality of life in patients with long-standing lateral epicondylosis.

**Methods:**

Eighty-one subjects with tennis elbow lasting for more than 3 months were randomly allocated to an exercise group (*n* = 40) or a reference group (*n* = 41). The exercise group performed daily exercise, with weekly load increase, for 3 months. The reference group was wait-listed, but otherwise followed in the same way. Outcome measures were pain during maximum voluntary muscle contraction (Cozen's test) and pain during maximum muscle elongation with a load (modified Empty-can-test); muscle strength was measured with a Chatillon MSE 100 hand-held dynamometer, and the Disability of the Arm, Shoulder and Hand (DASH) and the Gothenburg Quality of Life questionnaires.

**Results:**

The exercise group had greater and faster regression of pain, both during muscle contraction and muscle elongation, than the reference group (*p* = 0.0005 and *p* = 0.0016, respectively). There was a non-significant muscle strength difference between the groups, but no differences regarding DASH scores or quality of life measures.

**Conclusions:**

Exercise appears to be superior to expectation in reducing pain in chronic lateral epicondylosis.

## Background

Tennis elbow (TE) is a common disorder. Typical symptoms are pain at the lateral epicondyle of the humerus and pain on resisted dorsiflexion of the wrist (1). The incidence is estimated to be 1%–3% per year (2,3). Repetitive strain and heavy manual labour increase the risk of being affected (4). Most of the incidents heal within 3 months, but about one-third have a more protracted course, and an estimated 17% still have symptoms after 1 year (5).

The acute stage is dominated by inflammatory processes (6,7), which, through the release of prostaglandins and inflammatory peptides, may activate peripheral nociceptive neurons (8). This stage is accordingly termed epicondylitis or tendinitis (9). Rest and anti-inflammatory medication may be the proper treatment (6). If symptoms prevail for more than 3 months, the condition is labelled chronic (10). At this stage of disease, inflammatory cells are essentially absent, replaced by degenerative signs in the tissue (9,11,12), hence the suggested term epicondylosis or tendinosis (11,13). The aetiology of pain in the chronic stage is as yet unknown, although an increase of neural transmitters in the affected tissue has been found (14–19), which may be responsible for activating or sensitizing peripheral nociceptors (8). Uncertainty about the aetiology may explain why there is no clearly effective treatment in the chronic, tendinosis, stage of the disease (20).

A multitude of treatments have been proposed (21), many of which have not yet been properly evaluated (20,22). The common practice for treatment of chronic TE in primary care today is conservative treatment with rest and anti-inflammatory medication (23). Physiotherapy including exercise has been claimed to have better and faster effect (24,25), but due to the costs it has been argued that expectation is the most cost-effective treatment (25). A simplified protocol for exercise treatment of TE, requiring less utilization of health care resources, has been requested (26), possibly changing recommendations based on cost-effectiveness (25).

The aim of this study was to compare the effects of exercise according to a simple low-cost protocol versus expectation (wait-list) in chronic TE with pain, muscle strength, function, and quality of life measures as outcome.

## Study population and methods

### Study design

The study was performed in the city of Uppsala, Sweden, and nested in a larger long-term trial (‘main study’) comparing the effects of eccentric or concentric exercise. The present study was performed as a randomized controlled trial during 3 months of the effect of exercise, eccentric or concentric, versus being wait-listed on pain and muscle strength.

### Study population

For the main study all 150 general practitioners and 90 physiotherapists at primary health care in Uppsala County were asked for information on subjects with long-lasting TE problems. In addition, subjects with TE symptoms were invited to participate in a randomized controlled trial through advertisements in the main local newspaper in order to recruit a sufficiently large number of subjects. Based on analyses of the Tierp Health Care Database (27) approximately 140 cases fulfilling the inclusion criteria were expected in the catchment area.

Recruitment for the main study was performed by one of the authors (M.P.) from 15 October 2003 to 18 October 2006, and 33 patients referred from general practitioners, 16 from physiotherapists, and 62 recruited through advertisements were finally included. From 23 December 2004 consecutive subjects were assessed for participation also in the present study. Inclusion criteria were age 20–75 years, symptoms of TE for more than 3 months, and a verified diagnosis. Exclusion criteria were any of concomitant supinator syndrome, compartment syndrome of the anconeus muscle, rhizopathy, inflammatory joint disease, fibromyalgia, previous elbow surgery, and inability to understand Swedish.

**Figure 1.**
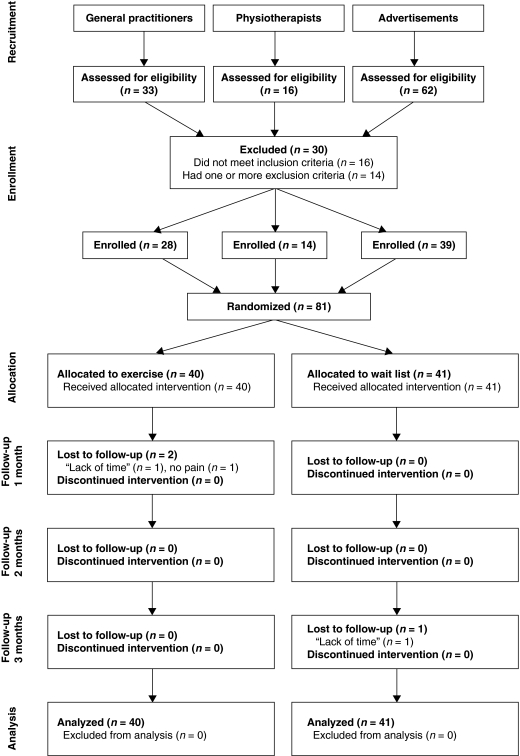
Flow chart of the study population.

At a first appointment the diagnosis was checked by pain on palpation, stretching (Mill's test), loading (maximum voluntary contraction (MVC)), and Maudsley's middle finger test (28) by the same physician, a general practitioner and pain specialist (M.P.). For a verified diagnosis, pain on palpation and a positive outcome of one or more of the other three tests was required. Of 111 subjects assessed, 81 satisfied all the inclusion and none of the exclusion criteria (Figure 1). Of these, 45 (55%) had their dominant arm affected, 25 (31%) the non-dominant arm, and 11 (14%) had both arms affected. All subjects gave written informed consent before entering the study. The Uppsala Regional Research Ethics Board approved the study.

### Randomization procedure

The subjects in the present study were randomly and blindly assigned by author K.S. to either an exercise group (*n* = 40) or a wait-list (reference) group (*n* = 41) by means of a random block design. The SAS ‘ranuni’ function, generating random numbers with equal probability distribution, was programmed so that for each consecutive four participants, two were randomly allocated to the exercise group and two to the reference group.

### Data collection

Data were collected at base-line and at follow-up visits at 1, 2, and 3 months after the base-line visit. At base-line, information was collected regarding educational level, marital status, smoking habits, TE history, and previous treatment given during the current episode. Education was classified on a four-degree scale ranging from compulsory education only to college or university education. Marital status was classified as never married, married or cohabiting, divorced, or widowed. Smoking habits were classified as never smoked, ex-smoker, currently smoking 1–14 cigarettes/day, 15–24 cigarettes/day, or 25 or more cigarettes/day (29). The TE history included number of previous episodes, time since last episode, and duration of the present one. Information on previous treatments during the current episode was given in a free format.

Pain reduction was the primary outcome of the study and measured at all visits with two 100 mm visual analogue scales (VAS) ranging from ‘no pain’ (=0) to ‘worst imaginable pain’ (=100). The first scale measured pain during MVC of the forearm extensor muscles (Cozen's test) (28,30). The second scale measured pain during maximum muscle elongation (MME) of the extensor carpi radialis brevis and longus muscles with a load (90° abduction of the arm followed by full pronation of the forearm with a 3-kg dumb-bell, i.e. a modified Empty-can-test) (28,30). Both pain measures were chosen in co-operation with an experienced hand surgeon to simulate the most accurate pain-provoking manoeuvres in tennis elbow. Based on the four measurements per subject across the study period, the coefficient of variation for pain during MVC, adjusted for the effect of time, was 16.7%, and for pain during MME, 12.5%.

The secondary outcome, muscle strength of the forearm extensor muscles, was also measured at all visits using a hand-held dynamometer (Chatillon MSE 100, AMETEK Inc., USA) using position and procedure as in the MVC pain score above. An analysis of repeated muscle strength measurements in three volunteers performed by three observers gave a coefficient of variation of 8.2% after adjustment for observer effect. This is in line with previous assessments of test–retest and inter-rater reliability concerning hand-held dynamometry (31,32).

The tertiary outcome, general arm function and quality of life aspects, were measured at base-line and at the 3-month follow-up visit, with the Disability of Arm, Shoulder, and Hand questionnaire (DASH) (33,34) and the Gothenburg Quality of Life Instrument (GQL) (35–37), respectively. DASH contains 30 questions on the ability to perform activities using a five-degree Likert scale ranging from ‘no problem’ to ‘impossible’. Responses were summarized and standardized so that the sum score, indicating overall degree of restriction, ranged from 0 to 100, low scores indicating a low degree of restriction.

GQL with its three sub-scales Complaint score, Well-being score, and Activity score has been validity-tested in various study populations and is widely used. The Complaint score lists 30 general symptoms. The respondents were asked to indicate which of these they had experienced during the last 3 months. Possible responses were ‘yes’ or ‘no’. The sub-scale is not intended to measure the presence of disease but the tendency to report complaints, an aspect of quality of life.

In the Well-being score, self-rated health was used. The respondents were asked to indicate their present situation on a seven-degree Likert scale ranging from ‘very bad’ to ‘excellent, could not be better’, with no verbal description of the intervening steps.

The Activity score lists 32 specified leisure time activities and two open alternatives, covering six areas. The subjects were asked to indicate which of these activities they had performed during the last year with response alternatives ‘never’ (0), ‘occasionally’ (1) and ‘often or regularly’ (2). The scores were summed across the area to an overall activity score, high scores indicating an active life-style.

### Intervention

**Figure 2.**
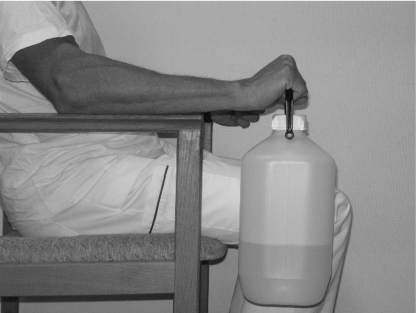
Photograph showing exercise set-up with the patient seated in an armchair with forearm support, holding the weight (a plastic container with a specified amount of water in it) in the affected arm, and performing exercise by lifting and lowering the container.

The reference group was informed that the condition was painful but harmless, that the arm should be used in ordinary daily activities, and the recommendation was to ‘wait and see’. The exercise group received the same information except that the recommendation to ‘wait and see’ was replaced with a 3-month daily exercise regime performed at home, with progressively increasing load on the extensor muscles of the affected forearm. The loading equipment consisted of plastic water containers with a handle. For the sake of simplified clinical application, the initial load was standardized to 1 kg (1 litre of water) for women and 2 kg for men. The participants sat in a chair and supported the forearm on the armrest or on an adjacent table. Holding the handle of the plastic water container with a clenched fist in pronation and the container hanging freely in front of the armchair or below the table-top (Figure 2), the load was lifted or lowered in three sets of 15 repetitions, 45 in total, once daily. The load was increased weekly by one-tenth of a kilogram (1 decilitre of water). The subjects were asked to report if competing treatment was given, but none reported such treatment. Subjects were instructed not to use pain-relieving or anti-inflammatory medication other than paracetamol. Adherence to instructions and the intervention programme was monitored. The same observer did all measurements. Since the observer also gave instructions about the exercise no blinded data collection was possible.

### Statistical considerations

Data were analysed using the SAS software, version 9.1. In the exercise group 93% participated in all follow-up visits and in the reference group 90%. In the exercise group 93% fully adhered to the exercise programme. Data loss owing to partial non-response (missing data in returned questionnaires and protocols) was 1%. The intention-to-treat approach was followed. The few missing data points were replaced with data from the nearest previous non-missing data measurement occasion.

For the main study an a-priori power calculation was done based on previous studies of chronic Achilles tendinitis and chronic tennis elbow comparing two active treatments. For the present study no a-priori power analysis was done since the length of the recruitment period was determined by the power analysis of the larger trial. However, a *post-hoc* power analysis for the present study showed 80% power for the pain variables with the actual study population size.

Simple differences between groups in continuous variables were computed with Student's *t* test and differences in proportions with the chi-square test.

The following analytical strategy was used. First, a crude data analysis was done based on differences between the base-line and the end of follow-up measurements in the exercise versus the reference group. Then an analysis was performed taking outcome measurements at all occasions into account, in order to compare temporal differences in pain regression and muscle strength improvement between the groups. In these analyses adjustments were made for outcome-affecting variables other than the exercise programme, such as age, sex, smoking habits, education, marital status, number of previous TE episodes, time since last episode, duration of the present one, and initial differences in the outcome variable, by including these as covariates in the analyses.

In the analyses of pain, muscle strength, DASH score, Activity score and Complaint score, all continuous variables, multiple linear regression was used. Since self-rated health is an ordinal variable, it was analysed with ordinal multiple logistic regression, as well as with multiple linear regression. However, the two methods gave the same results, and therefore only the results from the multiple linear regression analysis are shown. To avoid analysis model overload, non-significant covariates were excluded by backward elimination. The regression analyses provided adjusted mean values for each measurement occasion by treatment group. Moreover, adjusted mean values across the study period were used for statistical testing to provide optimum statistical power.

**Figure 3.**
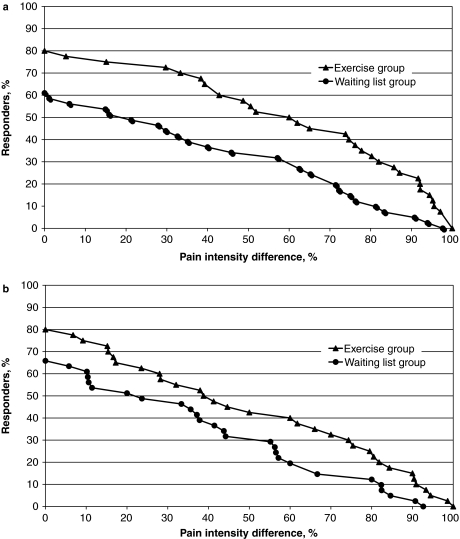
Cumulative proportion of responder analysis graph showing the proportions of subjects (vertical axis) that equal or exceed a specified improvement of pain during the 3-month treatment period (horizontal axis). The exercise group had higher responder rate at all levels of change in pain score during maximum voluntary contraction (A), as well as pain score during maximum muscle elongation (B).

As there are different opinions on what is a clinically meaningful pain reduction, a cumulative proportion of responder analysis was performed (38,39). For each individual level of pain reduction observed, the proportion of subjects that equalled or exceeded that level was calculated and plotted (Figure 3). This allows comparison between groups at any desirable cut-off point. The mean difference between the curves for the two groups represents the absolute risk reduction (ARR), which may be used to calculate the number-needed-to-treat (NNT = 1/ARR) in trials where the outcome variable is graded (39). All statistical tests were two-tailed. *P* values less than 0.05 were regarded as statistically significant.

## Results

### Base-line characteristics of the study population

Mean age was 48 years, somewhat more than 40% of the participants were women, almost half had a college or university education, 88% were married or cohabitating, and 5% were current smokers (Table I). The exercise group had an average of 1.3 previous TE episodes, range 0–20, 76 weeks on average since last episode, and a mean duration of the present one of 107 weeks (Table II). The corresponding data in the reference group was 0.8 previous episodes, 45 weeks since last episode, and 96 weeks' duration of the present episode.

**Table I.** Characteristics of the study population.

	Exercise group		Reference group		
	*n*	Mean (SD) or %	*n*	Mean (SD) or %	*P*
*n*	40		41		
Age, years		49.1 (8.1)		47.4 (8.6)	
Women, %	16	40.0	18	43.9	0.72
Educational level					0.99
Compulsory education only	3	7.5	2	4.9	
Vocational training	5	12.5	8	19.5	
Upper secondary school	12	30.0	12	29.3	
College or university	20	50.0	19	46.3	
Marital status					0.45
Never married	2	5.0	3	7.3	
Married or cohabiting	35	87.5	36	87.8	
Divorced or widowed	3	7.5	2	4.9	
Smoking habits					0.42
Never smoked	25	62.5	20	46.8	
Ex-smokers	12	30.0	19	46.3	
Current smokers	3	7.5	2	4.9	

SD = standard deviation.

**Table II.** Lateral elbow tendinosis history and previous treatments during the present episode.

	Exercise group		Reference group		
	*n*	mean (SD) or %	*n*	mean (SD) or %	*P*
Lateral elbow tendinosis history					
Number of previous episodes		1.3 (3.91)		0.8 (2.05)	0.48
Time since last episode, weeks		76.2 (202.14)		44.6 (142.34)	0.42
Duration of present episode, weeks		106.6 (192.7)		95.6 (118.8)	0.76
Previous treatments given					
NSAID	18	45.0	21	51.2	0.58
Acupuncture	15	37.5	13	31.7	0.59
Steroid injections	14	35.0	12	29.3	0.58
Stretching	10	25.0	11	26.8	0.85
Orthosis or other fixative	10	25.0	12	29.3	0.67
Manual treatment (deep friction, massage, manipulation)	6	15.0	8	19.5	0.59
Exercise	5	12.5	5	12.2	0.97
Rest	5	12.5	2	4.9	0.23
Ultrasound or laser	4	10.0	4	9.8	0.97
Other treatments	4	10.0	2	4.9	0.38

SD = standard deviation.

The most common previously given treatments during the present episode were, in rank order, non-steroid anti-inflammatory drugs (NSAID), acupuncture, steroid injections, stretching, orthosis or other supporting device, manual treatment, exercise, rest, and ultrasound or laser treatment. Most of the subjects had received some form of treatment. None of the base-line characteristics differed significantly between the exercise and reference groups.

### Analysis of crude outcome data

Crude outcome data are shown in Table III. The exercise group had a higher base-line level of the two pain scores and the DASH score, and lower muscle strength than the reference group, whereas the base-line levels of the Activity score, self-rated health, and Complaint score were similar. During the study period the exercise group had a larger crude decrease of pain during MVC (*p* < 0.01), pain during MME (*p* < 0.05), and a non-significant trend towards more muscle strength and larger decrease of the DASH score than the reference group. For the remaining outcome measures the differences in trend were small and of variable direction.

**Table III.** Crude outcome data by measurement occasion and treatment group.

	Exercise group				Reference group			
	Base-line	1 month	2 months	3 months	Base-line	1 month	2 months	3 months
Pain score, MVC	42.2 (26.5)	30.2 (26.1)	21.3 (22.1)	19.5 (21.1)	33.9 (29.3)	30.0 (29.2)	27.3 (28.7)	27.0 (27.9)
Pain score, MME	52.0 (21.5)	38.6 (29.2)	31.3 (26.2)	29.1 (25.9)	45.5 (27.8)	41.1 (27.9)	35.6 (27.5)	35.5 (26.7)
Muscular strength, N	130.4 (47.9)	129.2 (44.2)	141.3 (45.6)	137.7 (38.0)	141.1 (47.9)	138.2 (43.2)	144.0 (43.1)	140.9 (43.7)
DASH score	28.7 (12.8)	–	–	18.2 (14.6)	24.6 (14.7)	–	–	18.7 (14.9)
Activity score	29.1 (7.4)	–	–	28.0 (7.4)	28.0 (6.7)	–	–	27.9 (5.7)
Well-being	5.4 (1.3)	–	–	5.5 (1.3)	5.7 (1.3)	–	–	6.0 (1.0)
Complaint score	6.1 (4.1)	–	–	6.0 (5.3)	5.0 (5.2)	–	–	4.9 (4.6)

MVC = maximum voluntary contraction; MME = maximum muscle elongation.

The cumulative proportion of responder analysis for pain during MVC and pain during MME is shown in Figure 3. The exercise group had a higher responder rate at all levels of pain reduction, regardless of regression criteria, than the reference group. For instance, 72% of the subjects in the exercise group versus 44% in the reference group had 30% pain reduction or more during MVC. This represents an absolute risk reduction of 28% and a number-needed-to-treat of 1/0.28 = 4. The corresponding absolute risk reduction for MME was 15%, and number-needed-to-treat of 1/0.15 = 7.

### Analysis of outcome data adjusted for disturbing factors

In order to compare the change across time in the two groups as efficiently as possible, linear regression analyses utilizing measurements from all four measurement occasions were performed. Measured in this way the exercise group had a significantly lower level of pain during MVC (*p* = 0.0005) as well as during MME (*p* = 0.005) than the reference group. There was a non-significant trend towards a more favourable muscle strength and DASH score in the exercise group than in the waiting-list group (*p* = 0.17 and *p* = 0.30, respectively). No significant differences and no clear trends regarding any of the quality of life measures were found.

## Discussion

The exercise programme group had a significantly greater and faster recovery, in terms of pain during MVC and pain during MME, than the reference group. There was also a non-significant trend towards less restricted arm activity and arm muscle strength in the exercise group.

The strengths of the study include that the study population was recruited from among chronic tennis elbow patients in primary health care. The external validity versus this type of patients in general is supported by the fact that for the main study 140 cases were expected in the catchment area and 120 were found. Adherence to follow-up and the exercise programme were excellent; the data loss in the trial was low; and the same observer did all measurements, thereby avoiding inter-observer variation; and an intention-to-treat analysis strategy was used, thereby minimizing the risk of bias.

The limitations include that complete blinding, as in drug trials, was not possible in this type of intervention. A potential bias in non-blinded trials may be related to differences in expectations. As in all active treatment versus wait-list studies, subjects given active treatment may be presumed to have higher expectations of the treatment effects than wait-listed subjects, the latter perhaps having high expectations of the treatment-to-come, but not of any wait-list effect. The follow-up intervals were chosen to allow control of adherence to the programme. Preferably, the follow up would have been longer than 3 months. However, 3 months was as much we dared to delay active treatment in the wait-listed reference group, not to lose them as active participants in the main study.

Pain scoring using visual analogue scales (VAS) has previously been validated (40,41). The scoring has considerable inter-patient variability, but intra-patient variability over time, as used in this study, is low. Neither Cozen's test nor Empty-can-test has been tested for reliability and validity, but they are nevertheless considered gold standard in clinical practice (30). Muscle strength measurements with a hand-held dynamometer have reliable reproducibility in test–retest and between-day measurements (31,32). The DASH questionnaire has been recommended by the American Academy of Orthopedic Surgeons' Outcomes Research Committee and the Institute for Work and Health. The English and the Swedish versions have both been tested for reliability and validity (33,34). The Gothenburg Quality of Life instrument is a validated and extensively used measure of quality of life (35–37).

The largest differences between the groups were found in the two pain variables, as evaluated by the subjects themselves. The quality of life variables, especially self-rated health, may be anticipated to be more prone to expectation effects than pain or muscle strength. The fact that an effect on pain but not on quality of life was found favours the view that the treatment effect is not caused by differences in expectations to any major extent. The DASH measure was also subject-evaluated, but the difference between the groups was non-significant. The latter was unexpected, but in the context of a limited functional impairment, such as TE, DASH may be a somewhat insensitive measure (42). A more sensitive questionnaire for the specific evaluation of TE has since been developed (43).

To gain maximum effect of the exercise, the starting load should be individually tailored, for instance as percentage of one-repetition maximum (1 RM), the weight one can endure to lift once only (44). To simplify clinical application, the starting load in this study was standardized to 1 kg for women and 2 kg for men. This may have had the effect that the load, and accordingly the stimulus, in some individuals was smaller, or greater, than what would be required for optimum gain. Therefore, the effects of the exercise regime in this study may have been under-estimated.

Pain provocation measures often used to document symptoms in TE, such as pain during grip-testing or pain at rest, are non-specific for the muscles affected in TE, and validity is low. Specific movements that put stress on the affected muscles, tendons, and their insertions, provoke pain in TE, like in many other soft-tissue pain conditions. The outcome measures for pain used in this study were developed in co-operation with an experienced hand surgeon to be specific for the muscles affected in TE. MVC of the forearm extensor muscles (Cozen's test) puts maximum stress on the muscles involved in TE, i.e. extensor carpi radialis brevis, extensor carpi radialis longus, and extensor digitorum communis, which also connect to the tendinous insertion on the lateral elbow epicondyle. MME with a 3-kg dumb-bell (a modified Empty-can-test) simulates the manoeuvre most often described by TE patients as provoking everyday pain, such as lifting a frying pan or pouring out of a pot.

Recent years have seen a growing interest in exercise as treatment for chronic tendinopathies (24,45–47). A few recent studies have reported a clear tendency in favour of physiotherapy including exercise as compared with expectation (24,25). As compared with previous studies, our study is a more straightforward exercise versus wait-list trial and supports the idea that exercise is more effective than expectation in chronic TE.

The additional cost of active physiotherapy measures has been questioned (25) and a simplified exercise protocol for TE requested (26). The suggested exercise protocol used in this study is of a simple, low-cost kind that can be performed at home with a plastic container and an armchair. It does not require costly measures such as assistance of health care staff or specific exercise machines. We do, however, suggest one early follow-up appointment to confirm that instructions for exercise have been correctly understood. It may also boost patient motivation and compliance.

The human body has evolved to perform weight-bearing activities, and its function is dependent on regular physical activity interspersed with rest. Exercise promotes neural reorganization as well as hypertrophy of the muscle–tendon unit (44). Moderate mechanical stretching of the tendon, such as in a controlled exercise regime, will increase proliferation of stem cells inside the tendon (48). Activation of stem cells induces secretion of a variety of cytokines and growth factors that have both paracrine and autocrine activities (49). This may have a modulatory effect on nociception. Rest, on the other hand, reduces strength by reduction of muscle–tendon volume and neuromuscular capacity, as measured by electromyography, and has negative consequences for bone mineralization (44).

The implications of these findings are that a chronic soft-tissue pain condition such as chronic TE should not be treated with rest but with graded exercise. This is in line with other studies and with findings of pain psychologists, who point out the negative effects of inactivity and associated fear avoidance behaviour and suggest graded activity as a means of overcoming this problem (50). However, once physical function is restored, it should be noted that maintaining proper function is dependent on a balance between regular activity and regular rest (44). Hence, continued exercise of forearm extensors after rehabilitation is encouraged, but with reasonable weight and interspersed with rest.

In conclusion, the results of this study show that a specific graded exercise regime is more effective in reducing pain in chronic TE than a wait-and-see regime in a 3-month perspective. This suggests that a graded exercise regime may be of benefit in other chronic muscle–tendon pain conditions as well. The exercise was effective although it was performed according to a simple, standardized, low-cost, homeexercise protocol.
